# Dr. Mortimer M. Taplin, McGill

**Published:** 1913-10

**Authors:** 


### OBITUARIES.

*Readers are requested to report promptly the death of all physicians in Western New York, or former residents of this region, or graduates of any medical school in Western New York and to notify the families of the deceased of our desire to publish adequate obituary notices.*

---

Dr. Mortimer M. Taplin, McGill, 1892, died of pneumonia September 9 at the Rochester General Hospital, having continued to attend to his practice till two days before. He was born in Addison, Ontario, June 25, 1868. After graduating in medicine, he practiced with his uncle, Dr. O. O. Stowell of Copenhagen, N. Y., for four years, and then, in 1896, opened an office in Rochester. He was a member of the County and State Societies and of the Rochester Academy of Medicine and Pathologic Society. Having met Dr. Taplin in consultation and informally on several occasions, we can add a personal tribute to his sound judgment, personal charm and sterling qualities.

